# Supplementary material for: Short-term fasting of a single amino acid extends lifespan
Source: GeroScience. 2024 Feb 2;46(4):3607–15. doi: 10.1007/s11357-024-01078-3 (PMC11229437; doi:10.1007/s11357-024-01078-3)
Supplement: Supplementary file 1 — Supplementary file1 (DOCX 374 KB) [file 11357_2024_1078_MOESM1_ESM.docx]

## Supplementary materials

Supplementary Table 1. Ingredients in 1L of Sugar Yeast medium.

| Ingredient | Amount (L^-1^) | Supplier (order code) |
| --- | --- | --- |
| Sugar | 50g | Bundaberg Australia (M180919) |
| Autolysed Brewer’s yeast | 100g | MP Biomedicals (290331225) |
| Agar (grade J3) | 10g | Gelita Australia (A-181017) |
| Nipagin (10% w/v in 96% EtOH) | 30mL | Sigma Aldrich (W271004) |
| Propionic acid | 3mL | Merck (8.00605) |

Supplementary Table 2. Ingredients in 1L of complete synthetic medium.

| Ingredient | Amount (L^-1^) | Supplier (order code) |
| --- | --- | --- |
| L-arginine HCl | 0.814g | Sigma Aldrich (A5131) |
| L-alanine | 0.551g | Sigma Aldrich (A7627) |
| L-asparagine | 0.514g | Sigma Aldrich (A0884) |
| L-aspartic acid | 0.586g | Sigma Aldrich (A6683) |
| L-cysteine | 0.171g | Sigma Aldrich (C7477) |
| L-glutamic acid | 0.759g | Sigma Aldrich (G5889) |
| L-glutamine | 0.560g | Sigma Aldrich (G3126) |
| Glycine | 0.383g | Sigma Aldrich (G7126) |
| L-histidine | 0.327g | Sigma Aldrich (H8000) |
| L-isoleucine | 0.560g | Sigma Aldrich (I2752) |
| L-leucine | 1.020g | Sigma Aldrich (L8912) |
| L-lysine HCl | 0.682g | Sigma Aldrich (L5626) |
| L-methionine | 0.301g | Sigma Aldrich (M9625) |
| L-phenylalanine | 0.504g | Sigma Aldrich (P2126) |
| L-proline | 0.489g | Sigma Aldrich (P0380) |
| L-serine | 0.688g | Sigma Aldrich (S4500) |
| L-threonine | 0.552g | Sigma Aldrich (T8625) |
| L-tryptophan | 0.160g | Sigma Aldrich (T0254) |
| L-tyrosine | 0.460g | Sigma Aldrich (T8566) |
| L-valine | 0.599g | Sigma Aldrich (V0500) |
| Agar | 7.00g | Sigma Aldrich (A7002) |
| Sucrose | 17.12g | Sigma Aldrich (S1888) |
| Cholesterol | 0.3g | Glentham Life Sciences (GE0100) |
| Choline chloride | 0.05g | Sigma Aldrich (C1879) |
| Myo-inositol | 0.005g | Sigma Aldrich (I7508) |
| Inosine | 0.065g | Sigma Aldrich (I4125) |
| Uridine | 0.060g | Sigma Aldrich (U3750) |
| Thiamine | 0.0014g | Sigma Aldrich (T4625) |
| Riboflavin | 0.0007g | Sigma Aldrich (R4500) |
| Nicotinic acid | 0.0084g | Sigma Aldrich (N4126) |
| Ca pantothenate | 0.0108g | Sigma Aldrich (21210) |
| Pyridoxine-HCl | 0.0017g | Sigma Aldrich (P9755) |
| Biotin | 0.0001g | Sigma Aldrich (B4501) |
| Folic acid | 0.0005g | Sigma Aldrich (F7876) |
| CaCl_2_.2H_2_O | 0.250g | Sigma Aldrich (C7902) |
| CuSO_4_.5H_2_O | 0.0025g | Sigma Aldrich (C7631) |
| FeSO_4_.7H_2_O | 0.025g | Sigma Aldrich (F7002) |
| MgSO_4_ (anhydrous) | 0.250g | Sigma Aldrich (M7506) |
| MnCl_2_.4H_2_O | 0.001g | Sigma Aldrich (M3634) |
| Zn SO_4_.7H_2_O | 0.025g | Sigma Aldrich (Z0251) |
| KH_2_PO_4_ | 3.00g | Sigma Aldrich (P9791) |
| NaHCO_3_ | 1.00g | Sigma Aldrich (S8875) |
| Acetic acid (glacial) | 3mL | Merck (100063) |
| Propionic acid | 6mL | Merck (8.00605) |
| Nipagin (10% w/v in 96% EtOH) | 15mL | Sigma Aldrich (W271004) |

| Supplementary Table 3. Survival model of flies that were fed a complete diet during weeks 1, 2, 3, or 5 of age and then exposed to nicotine. Summary of cox-proportional hazards modelling with age as a continuous variable. Confidence level = 95% |
| --- |
| 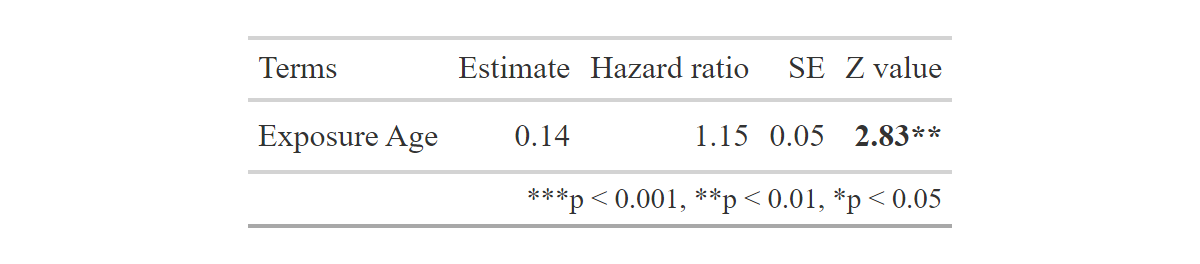 |

| Supplementary Table 4. Effects of duration of pre-treatment and age on the difference in survival between pre-treated flies and control flies. Summary of the linear model that best represented the relationship with duration of pre-treatment and age as continuous variables. Confidence level = 95% |
| --- |
| 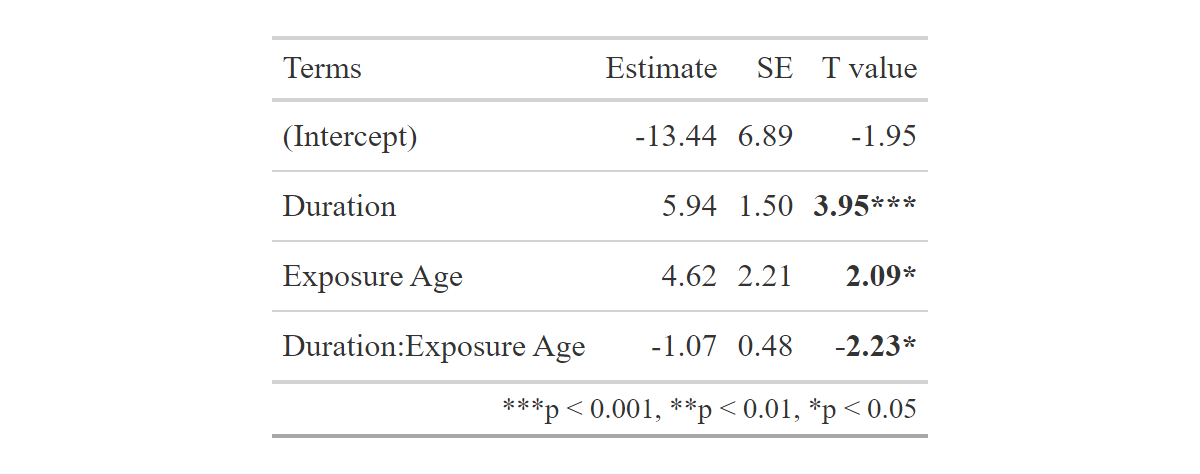 |

| Supplementary Table 5. Differences in survival between flies that were pre-treated with an isoleucine dropout for 1, 3, 5 or 7 days compared to flies that were fed a complete diet, separated by age when pre-treated. Summary of cox-proportional hazards modelling with duration of pre-treatment and age as discrete variables. Confidence level = 95% |
| --- |
| 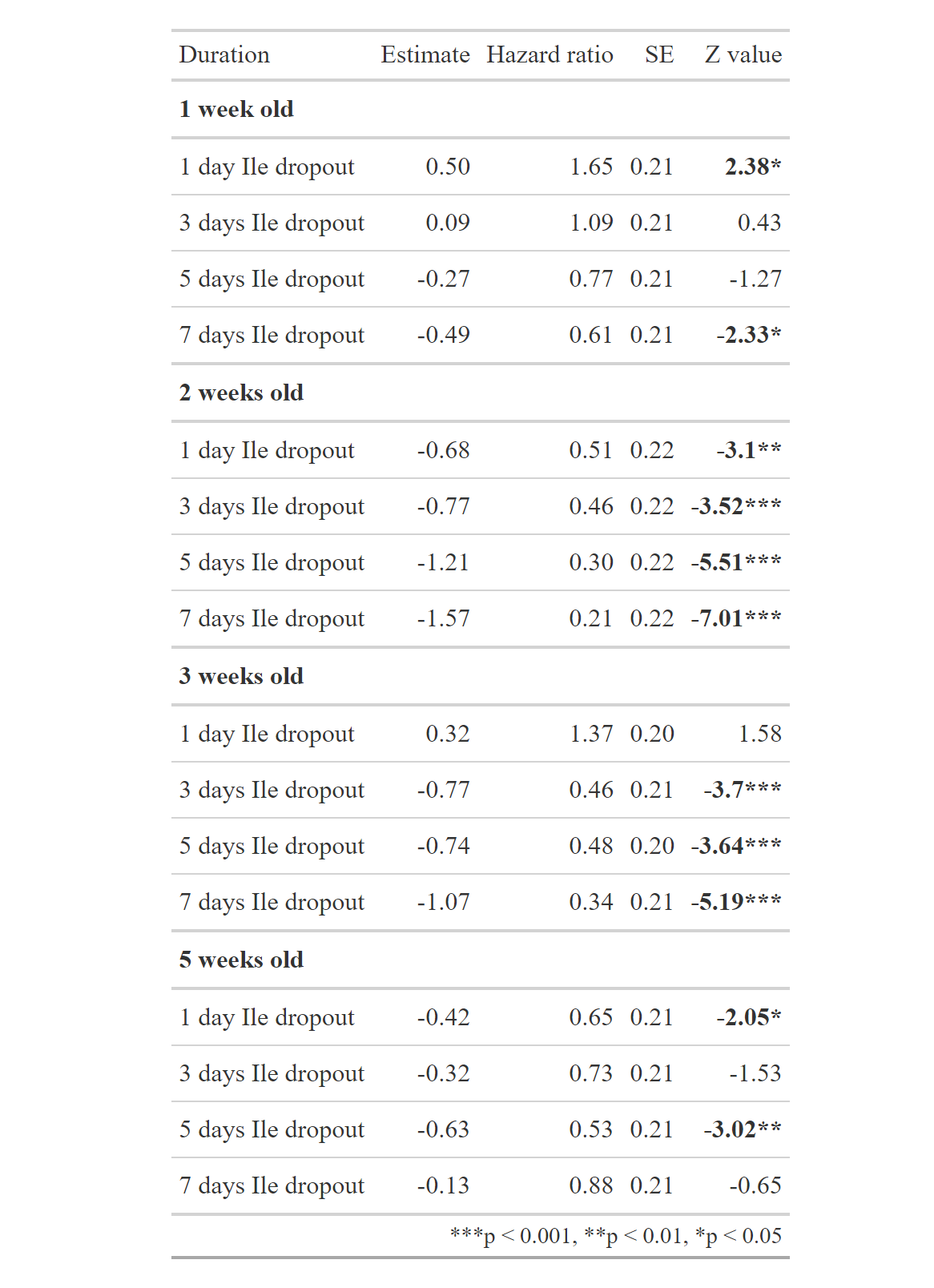 |

| Supplementary Table 6. Differences in survival between flies that were intermittently fasted of isoleucine and flies that were fed a complete diet for their lifespan. Summary of cox-proportional hazards modelling. The Terms column represents the sequence of Ile-deprivation treatments, where first is at 1 week of age, second at 3 weeks of age, and third at 5 weeks of age. Confidence level = 95%. |
| --- |
| 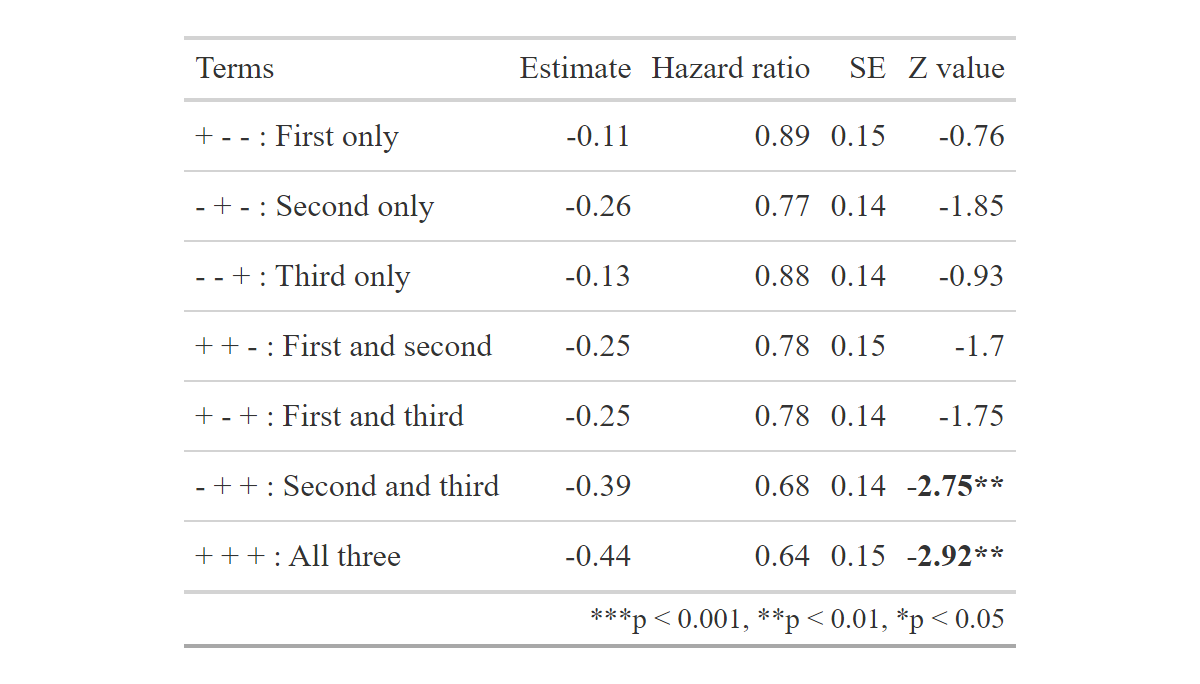 |

| Supplementary Table 7. The effect of returning isoleucine to the flies’ diet for 3 days before nicotine exposure on the difference in survival between pre-treated flies and control flies. The linear model was tested using emmeans^26^ to determine whether the difference in survival was different from the control (where control = 0 days). Confidence level = 95% |
| --- |
| 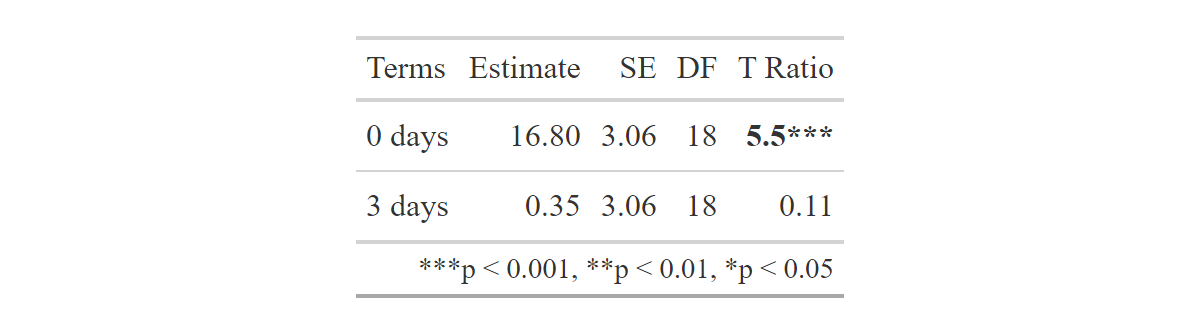 |

| 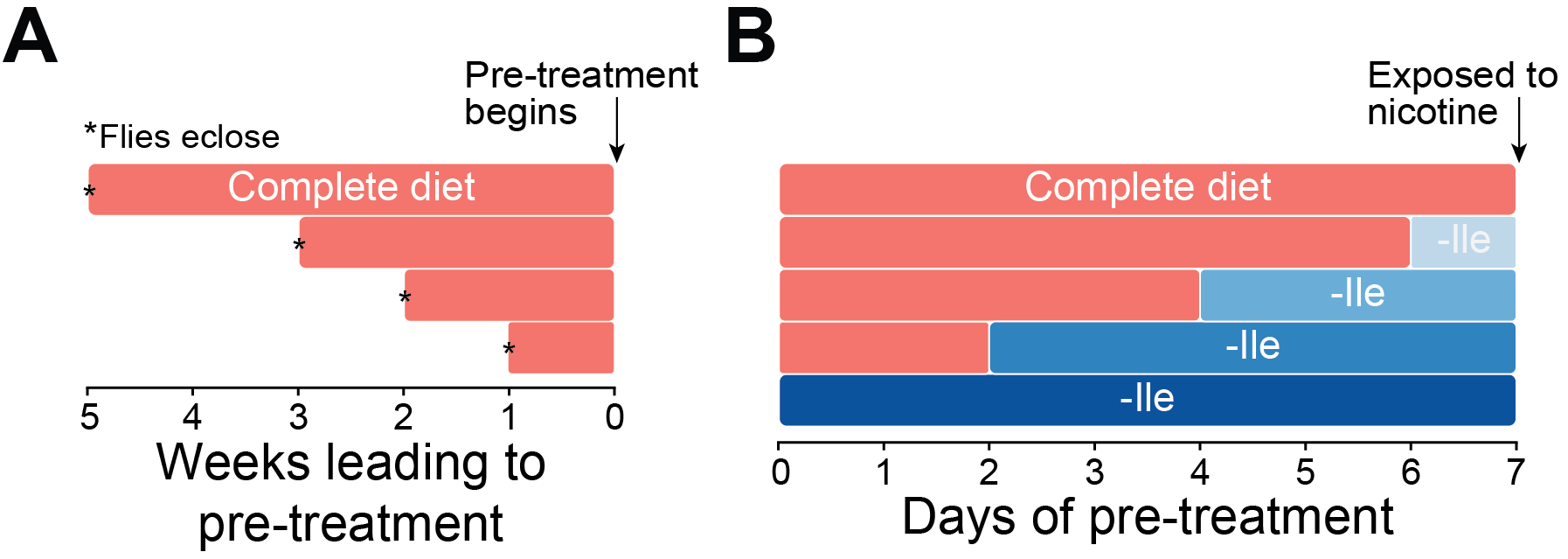 |
| --- |
| **Supplementary Figure 1. Methods depicting the treatment timeline for data shown in Figure 1**. (**A**) Controlled density egg lays were timed to ensure that flies eclosed at 5, 3, 2 and 1 week prior to the Ile-deprivation pre-treatment period. Flies were maintained on a complete, synthetic diet during this maintenance period. (**B**) During the pre-treatment period, flies were placed onto an isoleucine dropout for 7, 5, 3, 1 or 0 days immediately prior to nicotine exposure. Nicotine treatment was applied to all groups contemporaneously. |
